# A Rare Complication of Herpes Zoster Ophthalmicus (HZO)

**DOI:** 10.7759/cureus.35693

**Published:** 2023-03-02

**Authors:** Daniel Sen Kai Phang, Jaya Vani Ettikan, Hayati Abd Aziz, Francesca Martina Vendargon, Khairy Shamel Sonny Teo

**Affiliations:** 1 Ophthalmology and Visual Sciences, Universiti Sains Malaysia School of Medical Sciences, Kubang Kerian, MYS; 2 Ophthalmology, Hospital Sultanah Aminah, Johor Bahru, MYS; 3 Ophthalmology, School of Medical Sciences/Universiti Sains Malaysia, Kota Bharu, MYS

**Keywords:** magnetic resonance imaging, acyclovir, unilateral optic neuritis, herpes zoster ophthalmicus, hiv aids

## Abstract

Retrobulbar optic neuritis is a rare complication of herpes zoster ophthalmicus (HZO). We report a case of a 27-year-old man who presented with a progressive left blurring of vision for one week. A history of vesicular rashes in the left trigeminal nerve area preceded his condition. On examination, we noted that his left eye visual acuity was hand movement, and his optic nerve function was reduced. Findings from examining the anterior segment and intraocular pressure were unremarkable. The fundus examination results were normal. A blood investigation was positive for human immunodeficiency virus (HIV). MRI showed hyperintense features of the intraorbital segment of the optic nerve in the T2-weighted image. An abnormal high signal on a T2 weighted image may be present, which may be a clue for varicella zoster associated complications, such as HZO-related optic neuritis. Therefore, a diagnosis of retrobulbar optic neuritis was made, and antiviral treatment was initiated. He received two weeks of IV acyclovir and switched to the oral route for one month. After the completion of the treatment, his visual acuity remained the same.

## Introduction

Herpes zoster ophthalmicus (HZO) is caused by the varicella zoster virus (VZV) after reactivation from a dormant state. It involves the ophthalmic branch of the trigeminal nerve and can affect both the anterior and posterior segments of the eye. Retrobulbar optic neuritis is a rare complication of HZO, particularly in immunocompromised patients [1]. Reactivation of the virus occurs due to a decline in immune function, particularly the T-cell-mediated immune response in older patients or those with a history of taking immunosuppressive medicine [2]. It may coincide with vesicular rash or post-herpetic complications. The optic disc may initially appear normal or edematous, progressing to optic atrophy [1]. The prognosis of herpes zoster retrobulbar optic neuritis (HZON) is variable, ranging from complete disease resolution to severe visual loss despite being treated with antiviral drugs [3]. Here, we report a case of HZON in an immunocompromised patient treated with an antiviral medication.

## Case presentation

A 27-year-old man presented to the ophthalmology department, reporting concerns of progressive, painless blurred vision for three days. The blurring started in the inferior half of his vision which progressed to involve his central vision. Vesicular rashes preceded it over the left trigeminal nerve. It initially appeared on the upper left eyelid and extended to the forehead and scalp region. He also developed a low-grade fever but did not seek any medical treatment. He noticed that the rashes gradually improved with aqueous cream. His history was not suggestive of systemic or chronic disease. He had no history of drug abuse, promiscuity, or blood transfusion. His ophthalmic history was unremarkable.

**Figure 1:**
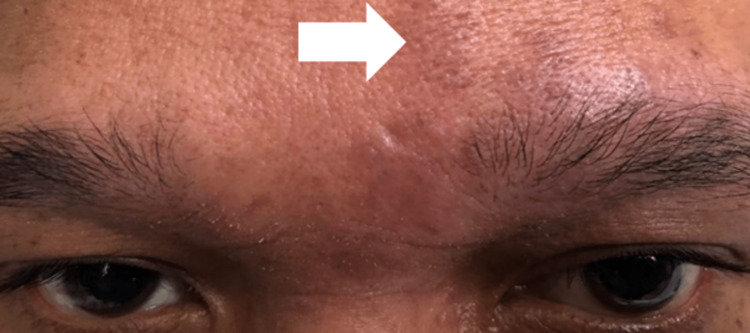
Vesicular rashes on the left eyelid and forehead region.

**Figure 2:**
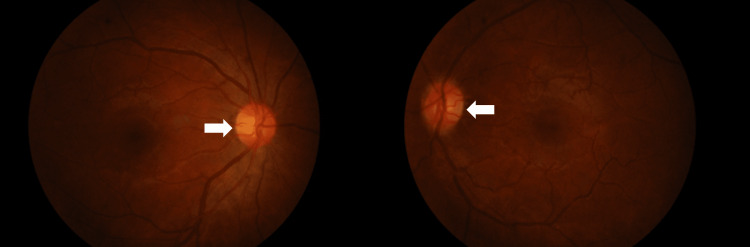
Fundus photo showing bilateral normal fundus.

Physical examination showed an alert, conscious, and well-built man. There was a vesicular rash in the left forehead region at the terminal nerve distributions (Figure 1). The best corrected visual acuity of the left eye was hand movement, while the best corrected visual acuity of the right eye was 6/12. There was a positive relative afferent pupillary defect and impairment of the optic nerve in the left eye. There was a significant reduction in light sensitivity and red desaturation to 90%, and his color vision test was also significantly affected. The Hutchinson sign was absent. Examination of the anterior segment and intraocular pressure of the left eye yielded unremarkable results. The fundoscopy examination findings were also normal, with no evidence of optic disc swelling (Figure 2). His right eye examination findings were unremarkable. A visual field assessment in the left could not be performed due to poor vision.

His laboratory investigation revealed a raised C-reactive protein level of 17.9 mg/dL. He was seropositive for the human immunodeficiency virus (HIV). Results from other blood tests, such as a complete blood count, erythrocyte sedimentation rate, and antinuclear antibody tests, were unremarkable. Serological tests for hepatitis B, hepatitis C, tuberculosis, and syphilis were all negative.

**Figure 3:**
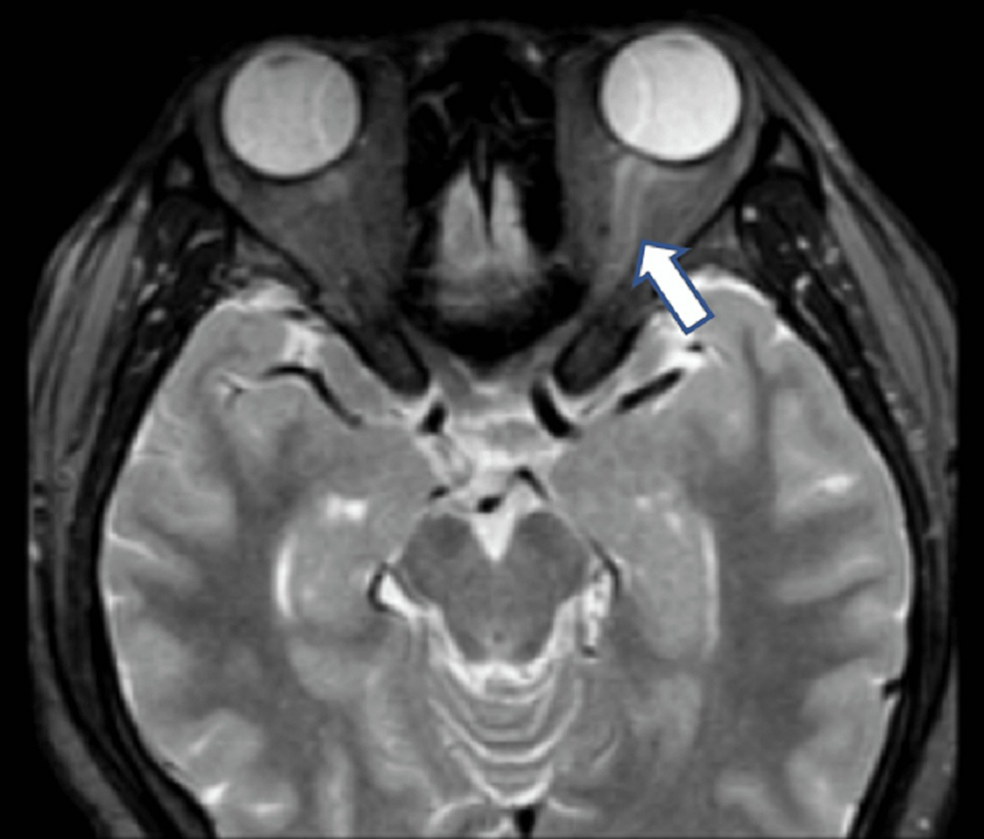
MRI of the orbit. Axial T2-weighted with gadolinium contrast showed enhancement (arrow) left optic nerve.

Magnetic resonance imaging with gadolinium contrast of the brain and whole spine showed a hyperintense intraorbital segment of the left optic nerve. The left optic nerve was bulky compared to the right optic nerve. There was minimal hyperintensity on the T2 weighted image at the retrobulbar region with no postcontrast enhancement. The right optic nerve was normal (Figure 3). 

A lumbar puncture was performed, and cerebrospinal fluid (CSF) was sent for analysis. The polymerase chain reaction test of his CSF was negative for cytomegalovirus, VZV, and herpes simplex virus. The CSF cell count, culture and sensitivity, acid-fast bacillus, and fungal staining were normal. There was no evidence of pleocytosis or elevated protein in the CSF.

**Figure 4:**
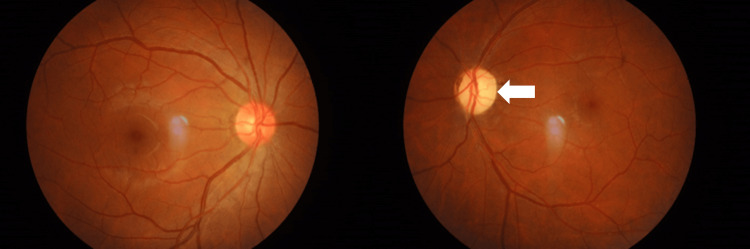
Fundus photo showing left optic atrophy.

Therefore, a diagnosis of left retrobulbar optic neuritis secondary to herpes zoster infection was made, and other causes of atypical optic neuritis were excluded. He received IV acyclovir 500 mg three times daily for two weeks, followed by oral acyclovir 800 mg five times daily for another two weeks. During treatment, there was no improvement in visual acuity or the optic nerve function test. Highly active antiretroviral therapy (HAART) was started four months later by the Infectious Diseases physician. His visual acuity remained hand movement at his one-year follow-up with evidence of left optic atrophy (Figure 4).

## Discussion

Herpes zoster infection is the result of the reactivation of the latent VZV in individuals who had varicella at some point in their lives. The virus remains latent in the dorsal root ganglia of sensory neurons. Reactivation occurs due to a T-cell-mediated decline in immune function, particularly the immune response, in older patients or those taking immunosuppressive medicine [2]. Our patient was immunocompromised, increasing the risk for reactivation of latent VZV. 

The ocular manifestation of herpes zoster occurs in 17% of cases [1]. It may affect the eyelid as vesicular rash, anterior segment (interstitial keratitis, disciform keratitis, or dendritic epithelial erosion), uveitis, secondary glaucoma, or posterior segment (acute retinal necrosis, progressive external retinal necrosis, vasculitis, optic neuritis) [1].

Optic neuritis is a rare complication of herpes zoster infection that can occur weeks to months after the onset of the rash. The onset of HZON occurs at a mean of 14.1 days (range, six to 30 days) after the initial appearance of the rash, which is consistent in our case. The degree of visual loss varies from mild to severe [3]. HZON is generally associated with other complications involving the anterior or posterior segment [1]. However, our case demonstrates an isolated HZON with no other ocular complications. Despite the normal CSF sample, a diagnosis of HZON was made given the laterality between the cutaneous manifestation of herpes zoster and optic neuritis, an MRI showing hyperintensity of the left optic nerve, and after excluding other possible causes of atypical optic neuritis [4].

The mechanism of optic nerve involvement is unknown. There are three proposed mechanisms of HZON [5]. First, one suggested mechanism is the direct extension of the virus through the cavernous sinus to the optic nerves and muscles. Another possibility is a local extension involving the meninges and brain, leading to meningoencephalitis that damages the optic nerve. The third proposed mechanism is generalized ocular ischemia secondary to inflammation [5].

Intravenous acyclovir remains the mainstay of the treatment of HZON. The recommended dose is 10 mg/kg three times daily for 10-14 days. There are no reliable data to support systemic corticosteroids [6]; 60% of patients who did not receive systemic corticosteroids showed improvement in visual acuity [3]. A study reported that only 25% of HZON patients responded to systemic corticosteroids. The duration of antiviral treatment varies from 10 days to two months. In general, visual acuity improves in four weeks [7]. In our case, we decided to continue treatment for up to one month due to his initial poor visual acuity on presentation. Treatment was stopped at one month, given that he had no significant improvement. The visual prognosis for HZON is usually very poor [5], and poor visual acuity in presentation is associated with poor final visual acuity [8]. Our patient's best corrected visual acuity at the presentation was hand movement. His final best corrected visual acuity after treatment did not improve.

The diagnosis of HZON should raise suspicion regarding the patient's immune status. Our patient had undiagnosed seropositive HIV. Immunocompromised patients with HZON should be treated early and aggressively. Four in 11 cases (36%) of immunocompromised patients with HZON had partial visual improvement with early treatment [9].

The initiation of HAART has been associated with VZV-associated vasculitis in the context of immune recovery uveitis [10]. The initiation of HAART can increase the risk of herpes zoster infection by two to four times from baseline in four to 16 weeks. HAART should be started 16 weeks after the initial presentation of HZON [11]. Our patient's HAART was started after four months, and the patient tolerated the medication well.

There are several reported cases of herpes zoster retrobulbar optic neuropathy that have been reported in the literature [3, 8, 12-13] (Table 1). Most of the cases are associated with poor visual outcome. In patient presenting with HZON, early detection and initiation of treatment can improve visual outcome [12]. 

**Table 1:** Summary of reported cases of herpes zoster retrobulbar optic neuropathy. Started before HZON onset refer to drugs that were initiated before and continued after the onset of optic neuropathy. Started after HZON onset refer to drugs that were initiated after the onset of optic neuropathy. PO, per os; BCVA, best-corrected visual acuity; CF, count fingers; HM, hand movement; NPL, no perception of light

Reference	Age/Sex	Time from rash to HZON (day)	BCVA at initial presentation for HZON	Other HZO manifestation	Treatment	BCVA at last follow up
Kaufman [3]	66/F	8	NPL	Iritis	Acyclovir 800 mg PO 5x/day for 9 days	NPL
Kaufman [3]	79/F	6	20/400	Iritis	Before HZON onset - topical vidarabine ; After HZON onset - oral prednisolone	NPL
Tunis [8]	19/M	27	NPL	Conjunctivitis	After HZON onset - oral prednisolone	CF
Singh [12]	58/M	4	20/120	Iritis	Started before HZON - oral acylorvir, topical acyclovir, and topical steroid	20/20
Schmidt [13]	73/F	28	LP	Epithelial keratitis	Before HZON onset - topical vidarabine; after HZON onset - topical prednisolone	20/600
Our case	27/M	7	HM	-	After HZON onset - IV acyclovir 10 mg/kg for 2 weeks then acyclovir 800 mg 5x/day for 2 weeks	HM

## Conclusions

In summary, we report a rare case of HZON which is a sight-threatening infection. Infective screening including HIV testing should be done in all patients presenting with retrobulbar optic neuritis. Optic neuritis should be suspected in a patient with herpes zoster who presented with an acute onset of blurring of vision. Prompt ophthalmology referral with early treatment initiation may improve visual outcome. 
